# Research on Detection and Tracking Technology of Cow Teats in Automatic Milking System Based on Lightweight YOLO-IWAL and Siamese Network

**DOI:** 10.3390/s26144584

**Published:** 2026-07-20

**Authors:** Jianfei Zhang, Huayu Zheng, Chengwei Jiang, Jiangjie Chen

**Affiliations:** School of Computer and Control Engineering, Qiqihar University, Qiqihar 161006, China; 2024912316@qqhru.edu.cn (H.Z.); 2023935765@qqhru.edu.cn (C.J.); 2025912329@qqhru.edu.cn (J.C.)

**Keywords:** cow teat detection, cow teat tracking, automatic milking systems, Time-of-Flight imaging, Siamese network, identity association, edge deployment

## Abstract

Automatic milking robots require accurate teat positions and consistent teat identities to guide cup attachment. However, weak texture, low contrast, rotated teat poses, and large inter-frame displacement in low-frame-rate Time-of-Flight (ToF) images make this task challenging, while embedded hardware imposes strict computational constraints. This study presents a cow teat detection and tracking framework comprising a lightweight YOLO-IWAL detector and a Siamese network-based tracking algorithm. Built on YOLO11n-OBB, YOLO-IWAL incorporates efficient feature extraction, detail-preserving downsampling, lightweight multi-scale feature fusion, and an oriented detection head. On the detection test set, YOLO-IWAL increases precision from 99.49% to 99.69%, reduces the number of parameters from 2.65M to 1.85M and computational complexity from 6.6 to 3.4 GFLOPs, and improves inference speed from 1039.2 to 1556.9 frames per second compared with the baseline. The Siamese network combines teat appearance features with 3D spatial information through Fourier positional encoding, adaptive geometric encoding, and gated fusion, and then applies the Hungarian algorithm for inter-frame identity association. On the tracking test set, it achieves an inter-frame identity association accuracy of 74.59% and an identity F1 score of 93.24%. Edge deployment on the RK3588 platform achieves 22.9 frames per second for YOLO-IWAL and a tracking processing speed of 166.39 frames per second for the INT8-quantized Siamese network. Overall, the proposed methods provide an efficient perception solution for cow teat detection and cross-frame identity association in automatic milking robots.

## 1. Introduction

Large-scale dairy farming has increased the demand for automated milking technologies that can reduce labor dependence and improve operational consistency. Automatic milking systems (AMSs) have become an important technological route for reducing manual labor in dairy production [1]. These systems rely on robotic manipulation combined with sensing technologies to guide milking-cup attachment [2]. During automatic cup attachment, the perception module must continuously provide accurate teat positions and stable teat identities for robot control. Localization errors or identity mismatches can directly cause attachment failure. Achieving reliable teat perception under practical milking conditions remains challenging. Milking parlors introduce illumination changes, udder or leg occlusion, hair and dirt interference, and body movement, all of which can affect teat visibility and localization. Moreover, teat motion and morphological variation during milking further increase the difficulty of maintaining real-time localization and identity consistency. Although 2D/3D sensing and deep learning techniques have been explored for cow teat detection and localization [3,4], many studies still emphasize single-frame teat localization or are validated mainly under laboratory conditions. Practical automatic milking, however, requires continuous perception: the system must locate teats accurately and maintain consistent teat identities across consecutive low-frame-rate ToF frames, as well as operate on resource-constrained edge hardware. Existing teat tracking research has mainly relied on conventional visual tracking techniques such as SURF-KCF, but validation under practical farm conditions remains limited [5]. Meanwhile, general tracking-by-detection methods are not specifically adapted to highly similar teat appearances, weak texture, and large inter-frame displacement in ToF amplitude images. Therefore, automatic milking robots still require a lightweight perception method that can perform oriented teat detection, maintain cross-frame identity association, and operate efficiently on edge hardware.

To address this need, this study develops YOLO-IWAL, a lightweight detector based on YOLO11 for cow teat detection in automatic milking robots, and combines it with a Siamese network-based cow teat tracking algorithm. Together, these two modules provide a perception framework for detecting and tracking multiple teats in dynamic milking scenes. Specifically, the main contributions of this study are summarized as follows.

1. Based on YOLO11n-OBB, we developed YOLO-IWAL for cow teat detection in ToF amplitude images acquired from automatic milking systems. While maintaining detection accuracy, the model introduces an efficient feature extraction network, lightweight multi-scale fusion, and an attention mechanism, significantly reducing the number of parameters and computational cost. This balance between accuracy and computational cost makes YOLO-IWAL suitable for real-time perception on low-performance embedded hardware in automatic milking systems.
2. A cow teat tracking algorithm based on a Siamese network is established. It integrates a MobileNetV3-Small backbone, Fourier positional encoding, an adaptive geometric encoder, and a gated fusion mechanism to generate discriminative normalized identity vectors. An association cost matrix is constructed from the distances between normalized identity vectors in adjacent frames, and the Hungarian algorithm [6] is then applied to achieve inter-frame teat identity association.
3. YOLO-IWAL and the Siamese network-based tracking algorithm were separately deployed and evaluated on an ARM-based RK3588 development board. The experimental results demonstrate the feasibility of real-time teat detection and identity association on resource-constrained edge hardware.

## 2. Related Work

### 2.1. Cow Teat Detection

In automatic milking systems, rapid and accurate cow teat detection from images is a key prerequisite for robotic cup attachment. Compared with general livestock detection tasks, cow teat detection is more challenging because teats are slender, weakly textured, low in contrast, and often affected by udder background interference, hair occlusion, and illumination variation. In addition, teat structures, including teat length and thickness, may change during milking [7], further increasing the difficulty of stable localization.

To address teat detection and localization, several vision-based methods have been explored in automatic milking research. Azouz et al. [3] developed a multimodal vision system for teat sensing by integrating optical stereovision and thermal imaging and validated its ability to locate the 3D coordinates of four teats within one second using a dummy thermal udder. Rastogi et al. [8] developed Haar-like feature-based and YOLO-based teat detection methods and compared their detection performance on a silicone teat model under indoor conditions. Overall, these studies demonstrated the feasibility of different visual teat detection methods, but their experimental settings were relatively idealized, relying mainly on artificial teats, indoor environments, or laboratory milking devices, and were not validated in real automatic milking environments.

Lu et al. [9] established an udder image dataset under the working environment of a rotary milking system, annotated cow teats using rotated bounding boxes, and developed an R2 Faster R-CNN-based teat detection model that could detect overlapped or partially occluded teats. Yu et al. [4] proposed FS-YOLOv4 for cow teat detection in milking parlors and evaluated it on real cow teat images collected from a dairy farm, achieving 98.26% mAP and a detection time of 26 ms. Although these methods demonstrated strong detection performance on real milking-scene images, their studies mainly focused on detection accuracy, while quantitative evidence related to edge deployment, such as parameter count, FLOPs, model size, and inference performance on embedded hardware, was insufficiently reported. Therefore, the edge-deployment feasibility of cow teat detection models for resource-constrained automatic milking robots still requires further investigation.

In summary, although existing studies have achieved promising progress in cow teat detection, they still face limitations in real-environment adaptability, model lightweightness, and edge deployment efficiency. Therefore, a lightweight and robust teat detection model is still needed for accurate and real-time teat localization in practical automatic milking systems.

### 2.2. Cow Teat Tracking

In practical milking operations, dairy cows are not stationary; even with restraining devices, teat motion persists throughout the process. Such displacement can cause misalignment between the robotic cup unit and the teat, leading to attachment failures. Therefore, real-time and high-precision teat tracking and localization are essential for automatic milking systems, and they impose high requirements on computational efficiency and robustness. Zheng et al. [5] proposed a SURF-KCF-based method that combines SURF feature detection with FLANN matching to enable autonomous target identification in the KCF framework. A scale-pyramid strategy was further used to adaptively adjust the tracking box size, thereby improving stability in dynamic scenes. Experiments conducted on a 1:1 laboratory cow model and Simmental cattle demonstrated accurate teat localization under slight motion, illumination variation, and partial hair occlusion, and the system successfully guided a robotic arm to complete cup attachment through coordinate transformation. However, this method has not been sufficiently validated under real farm conditions and does not fully consider teat contamination, complex lighting, dense environmental disturbances, simultaneous multi-teat tracking, or long-duration stability.

Generic tracking-by-detection methods provide useful association frameworks, but they are not fully matched to the sensing conditions of automatic milking. SORT [10] relies on motion prediction and temporal continuity, while IoU-Greedy [11] mainly depends on box overlap for data association. DeepSORT [12], ByteTrack [13], BoT-SORT [14], and OC-SORT [15] further improve association through appearance cues, detection confidence, or motion compensation. However, ToF amplitude images are grayscale-like intensity images and do not contain the rich color and texture information commonly used by general Re-ID models. In addition, cow teats within the same udder are similar in shape and appearance, and their cropped regions often contain weak texture and low contrast. Under low-frame-rate ToF imaging, adjacent-frame teat boxes may also show little spatial overlap because of cow body movement and teat wobbling. These factors weaken both appearance-based Re-ID features and geometry-only association, motivating the Siamese network-based tracking method that combines teat appearance features with 3D spatial information.

## 3. Dataset

Before constructing the datasets, we searched the relevant literature and public dataset repositories, including Roboflow Universe, Zenodo, Kaggle, and Google Dataset Search. Although several public resources related to cow udders or teats were found, they were mainly RGB image datasets for object detection or keypoint detection and did not provide synchronized ToF amplitude images, depth maps, oriented bounding box annotations, and cross-frame identity labels. Lu et al. [9] established an udder image dataset under a rotary milking system environment and used rotated bounding boxes for teat detection; however, this dataset was mainly used for single-frame detection and was not released as a public tracking dataset.

### 3.1. ToF Imaging System and Data Acquisition

ToF cameras, a type of 3D imaging device, calculate distances using the known speed of light to acquire depth information. Typically, a ToF camera system emits a wave signal in the near-infrared (NIR) spectral range, detects the reflected signal via a sensor, and measures the phase difference between the transmitted and reflected waves. Using the known wave frequency and the measured phase shift, the ToF camera can derive the depth information of the target object. To obtain spatial data for the automatic milking robot system, a 3D ToF camera O3D313 manufactured by IFM electronic was employed. This is a mature and reliable industrial-grade camera with an IP 69K protection rating, which meets the waterproof and corrosion-resistant requirements under milking scenarios involving spraying and cleaning processes. The camera features a resolution of 352 × 264 pixels, an operating range of 0.3 m to 8 m, and a default lens with a field of view (FOV) of 60° × 45°. It is connected via an Ethernet RJ45 interface and supports real-time 3D imaging at a maximum frame rate of 25 frames per second (FPS) under ideal settings. During data acquisition, the actual operating frame rate was approximately 8 FPS. This reduction was mainly caused by the camera-side acquisition configuration used to obtain stable ToF amplitude and depth images in the milking environment, including exposure settings, measurement range, filtering, and depth-related processing. Therefore, the 8 FPS value reflects the practical acquisition rate of the ToF camera under the selected milking-scene imaging configuration.

The O3D313 ToF camera was mounted in a fixed position on the robotic arm of an automatic milking robot. Data collection was conducted at a commercial dairy farm located in Daqing City, Heilongjiang Province, China, from March 2025 to April 2025, encompassing diverse scenarios to simulate real-world operational dynamics: images were captured at multiple angles, distances (to replicate dynamic tracking processes), and time periods (to account for varying illumination conditions). During data acquisition, the ToF amplitude images and depth maps were synchronously stored in H5 format, while the robot arm pose information at the corresponding timestamps was recorded in TXT files. A total of 11,072 ToF amplitude images of cow udders were initially acquired. For the tracking task, continuous ToF video clips were also recorded during the process from cow entry into the milking station to cup attachment, including natural cow movement and stress-induced body shaking during practical milking operations.

### 3.2. Detection Dataset Construction

After data acquisition, manual screening was conducted to exclude images without visible teats or with excessive blurriness that rendered annotation infeasible. A unified labeling protocol was then followed during annotation. Four annotators used the open-source CVAT [16] annotation tool to annotate each visible cow teat with an oriented bounding box, and the annotated data were exported in the YOLO-OBB format. The long axis of each oriented bounding box was aligned with the main direction of the teat, and the box boundary was adjusted to tightly cover the visible teat region as much as possible. After the initial annotation, the annotators exchanged the labeled data for manual cross-review. Ambiguous, severely blurred, or inconsistent annotations were corrected or removed to ensure labeling consistency. Finally, 9032 annotated images were split into training, validation, and test sets at a ratio of 7:1:2. A relatively larger proportion of the test set was allocated to more comprehensively evaluate the effectiveness of the proposed model improvements and its generalization capability on unseen data, ensuring fair performance comparisons across different models.

### 3.3. Tracking Dataset Construction

Unlike the detection dataset, the tracking dataset further required consistent identity labels for each cow teat across consecutive frames. During annotation, the numbers 1, 2, 3, and 4 were used to represent the identity IDs of the corresponding teats across frames. Taking the frontal view of the cow as the reference, IDs 1, 2, 3, and 4 corresponded to the right-rear, left-rear, right-front, and left-front teats, respectively. These anatomical identity labels were kept consistent across consecutive frames for tracking annotation. After annotation and manual review, a script written in Python 3.10.12 was used to calculate the 2D endpoint of each teat in every image. Specifically, for each OBB annotation, the midpoint of the short edge closer to the distal end of the teat along the major axis was defined as the 2D teat endpoint. The 3D information of each teat endpoint was then obtained by combining the 2D endpoint with the corresponding depth information and the robot arm pose recorded at the same timestamp.

**Figure 1:**
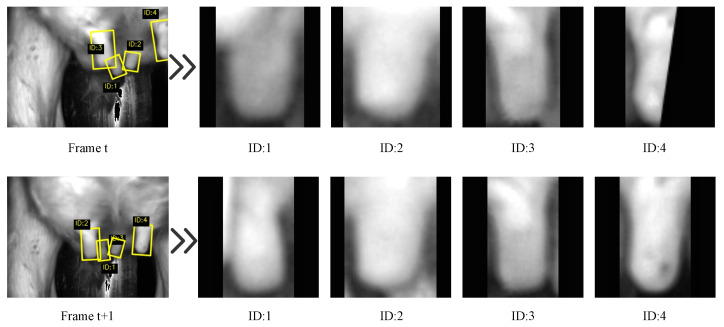
Annotated ToF frames, teat crops, and identity labels in adjacent frames. The displayed crops are contrast-enhanced for visualization only.

For Siamese network training and evaluation, individual teat samples were generated from adjacent ToF frames. Specifically, each teat region was cropped from the original ToF amplitude image according to the OBB annotation, rectified by perspective transformation, resized with aspect-ratio preservation, padded with black pixels, and standardized to 224×224 pixels. Meanwhile, the 3D coordinate of the corresponding teat endpoint was retained for spatial feature construction. Based on adjacent frames, teat samples with the same identity ID were defined as positive pairs, whereas samples with different IDs were defined as negative pairs. Figure 1 illustrates annotated ToF amplitude frames and the corresponding perspective-rectified teat crops with identity labels in two adjacent frames. To closely reproduce practical milking conditions, the collected video clips include severe motion caused by cow stress responses and body shaking, providing challenging samples for evaluating tracking robustness under low-frame-rate ToF imaging and large inter-frame teat displacement.

Overall, the complete tracking dataset covers 47 cows, 766 video clips, and 3770 frames. Based on these data, 39,879 cross-frame pairs were generated, including 10,554 positive pairs and 29,325 negative pairs. Using a fixed random seed of 42, the tracking dataset was partitioned at the cow level before cross-frame pair construction, resulting in 28 cows for training, 12 cows for validation, and 7 cows for testing. All video clips from each cow were assigned exclusively to one of the three subsets. The resulting subsets contained 460, 191, and 115 video clips, respectively. Cross-frame pairs were generated separately within each subset, resulting in 24,560 training pairs, 9469 validation pairs, and 5850 test pairs. Therefore, no cows, video clips, source frames, cropped teat images, or cross-frame pairs overlapped among the three subsets.

## 4. Proposed Method

### 4.1. Overall Detection and Tracking Framework

**Figure 2:**
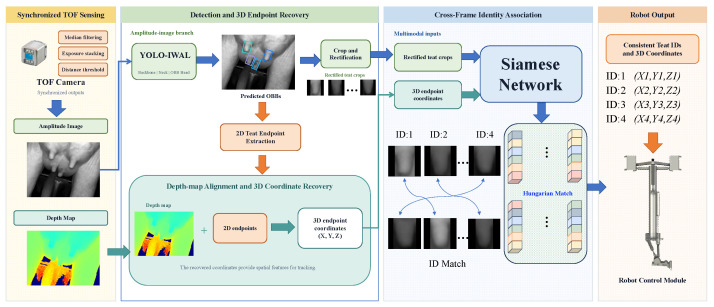
Schematic diagram of cow teat detection and tracking pipeline based on improved YOLO-IWAL and the Siamese Network.

To ensure the reliability of automatic milking robots during end-effector operations, the precise, stable, and continuous estimation of cow teat positions is critical. To this end, this study constructs a dynamic detection and tracking framework integrating an improved YOLO11 and a Siamese network and simultaneously incorporates ToF depth imaging to achieve accurate 3D localization of cow teats in milking scenarios. Our methodology combines multi-modal perception, enhanced detection, robust tracking, and 3D localization modules as shown in Figure 2. The framework takes ToF amplitude images and depth maps acquired by a ToF camera as inputs and comprises image preprocessing, object detection, Siamese feature extraction, cross-frame association, and depth-map alignment for 3D endpoint recovery. The overall procedure can be divided into two main stages: cow teat detection and cross-frame teat tracking.

**Figure 3:**
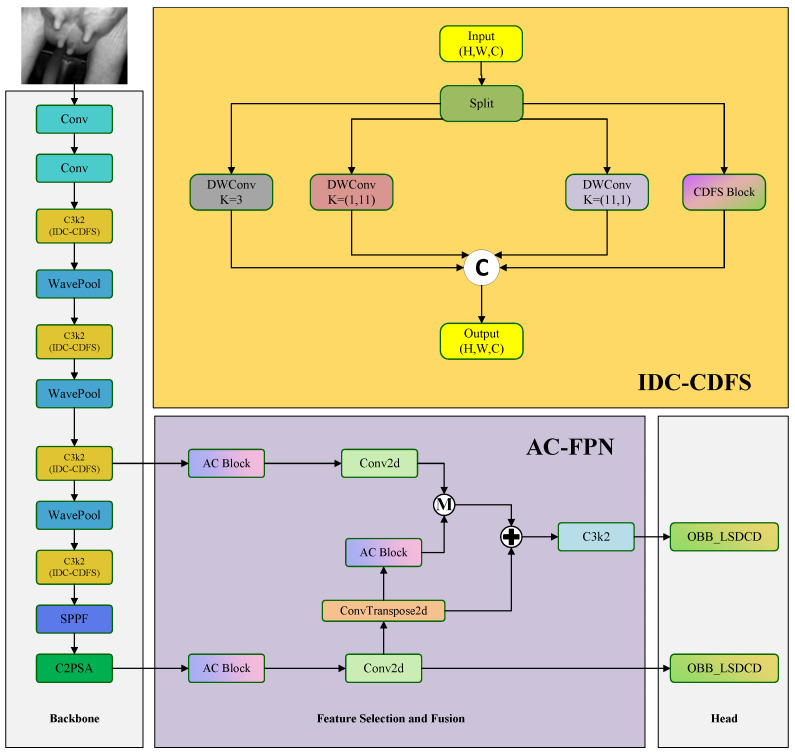
Network structure diagram of YOLO-IWAL. C and M denote concatenation and element-wise multiplication, respectively.

In the detection stage, cow teat detection is accomplished by the improved YOLO-IWAL model. Considering the real-time requirements of automatic milking robots and the limited computing resources of embedded industrial computers, YOLO11n-OBB is adopted as the base detector and further improved for lightweight oriented teat detection. Compared with horizontal bounding boxes, oriented bounding boxes can more tightly describe slender and rotated teat targets, reducing irrelevant background interference during training. To adapt to the elongated shape, weak texture, low contrast, and frequent occlusion of teats in ToF amplitude images, YOLO-IWAL introduces IDC-CDFS and Wavelet Pooling to enhance efficient feature extraction and detail-preserving downsampling. According to the bounding-box scale distribution of the dataset, AC-FPN removes the redundant P3 branch and screens informative features from the remaining multi-scale layers for efficient feature fusion, thereby reducing high-resolution prediction and post-processing overhead. OBB_LSDCD is introduced as a lightweight detail-enhanced detection head to strengthen high-frequency gradient and directional boundary representation for weak teat edges and rotated contours while maintaining inference efficiency. In this way, YOLO-IWAL provides lightweight and accurate OBB proposals for subsequent teat endpoint extraction, 3D coordinate recovery, and Siamese network-based tracking. The network architecture of YOLO-IWAL is illustrated in Figure 3.

**Figure 4:**
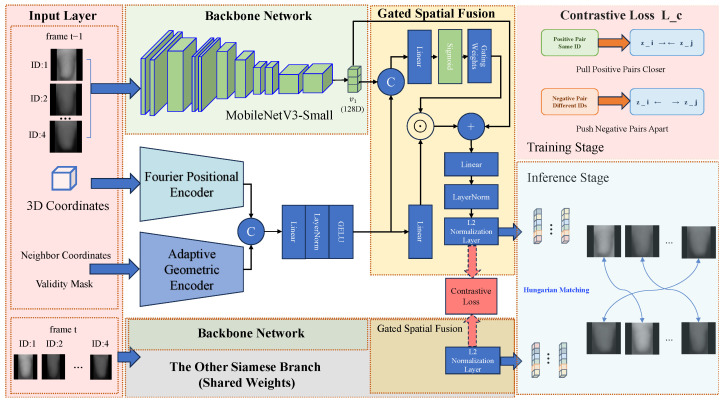
Architecture of the Siamese network-based tracking algorithm for cow teat tracking.

In the tracking stage, to address the large inter-frame displacement caused by the low frame rate of the ToF camera and irregular teat wobbling, this study proposes a Siamese network-based feature matching method for cow teat tracking. Unlike IoU-based association methods, this method does not rely on spatial overlap between adjacent bounding boxes. Instead, it learns deep appearance features from teat crops and incorporates 3D endpoint coordinates obtained from the ToF depth map, measuring teat similarity in the fused feature space to achieve reliable identity association under low-frame-rate conditions. The Siamese network consists of four main components: a visual encoding backbone, a Fourier positional encoder, an adaptive geometric encoder, and a gated fusion mechanism. The visual backbone extracts appearance features from cropped teat images, while the Fourier positional encoder and adaptive geometric encoder transform 3D endpoint coordinates into spatial features. The gated fusion mechanism then integrates the appearance and spatial features to generate normalized identity vectors. During training, contrastive loss is used to minimize the distance between the identity vectors of positive pairs while enforcing a predefined margin between those of negative pairs. During inference, the normalized identity vectors of teats in adjacent frames are used to construct an association cost matrix, and the Hungarian algorithm is applied to obtain the optimal inter-frame identity assignment. The Siamese network model used in this study is shown in Figure 4.

### 4.2. YOLO-IWAL

#### 4.2.1. Convolutional Module Improvement IDC-CDFS

The YOLO11 network relies on standard convolution as its fundamental operator for feature extraction, enabling efficient capture of local texture and edge information. However, single-layer configurations suffer from high computational costs and limited receptive fields, necessitating multi-layer stacking to achieve global feature representations. This approach, while deepening the network structure, introduces additional computational and storage overheads.

**Figure 5:**
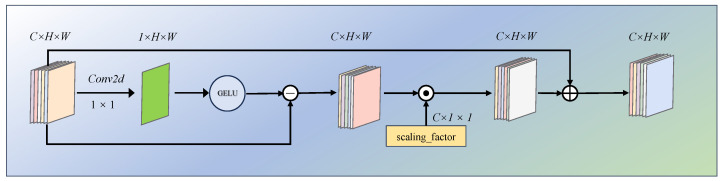
Network Architecture Diagram of CDFS Block.

In cow teat detection tasks, targets exhibit significant scale variations and low contrast with the background. Traditional standard convolution demonstrates limitations in directional sensitivity and multi-scale modeling, failing to fully characterize the longitudinal structural features of teats and effectively distinguish them from similar regions. These shortcomings ultimately constrain the detection accuracy and robustness of the model. The C3k2 module in YOLO11 inherits the advantages of the C2f module from YOLOv8 by integrating shortcut connections and feature splitting. This design enhances feature expression capabilities and gradient propagation efficiency while maintaining a lightweight architecture. Building on this foundation, we further optimize the C3k2 structure by replacing standard convolutions in the Bottleneck with Inception Depthwise Convolution (IDC) [17], inspired by the InceptionNeXt architecture. IDC advances the Inception module of GoogLeNet by adopting depthwise separable convolutions to replace traditional multi-scale convolutions. This decomposition of conventional convolutions into depthwise and pointwise convolutions significantly reduces parameter counts and computational complexity while preserving the model’s expressive capability and performance. This structure is particularly suitable for resource-constrained scenarios such as mobile and embedded devices. Furthermore, we introduce the Channel Decomposition and Feature Scaling (CDFS) mechanism to the IDC framework, proposing the IDC-CDFS module. This enhancement aims to further improve model efficiency and accuracy by leveraging channel-wise decomposition and adaptive feature scaling. Specifically, we used the CDFS Block to replace the identity branch in the original IDC of the second module within the Bottleneck of C3k2 in the backbone network. The network architecture of the proposed CDFS Block is illustrated in Figure 5.

These modifications effectively reduce parameter counts and computational overhead without compromising detection accuracy. Specifically, IDC and CDFS are employed to replace Conv layers in the Bottleneck, achieving parameter and computational efficiency gains without sacrificing performance. The original Inception module improves computational efficiency and reduces parameters by introducing parallel structures and convolutional kernels of varying sizes to extract multi-scale features. This strategy aggregates sparse matrices into relatively dense submatrices. Additionally, auxiliary classifiers are integrated to facilitate model fusion and amplify backpropagation signals. In our improved modules, asymmetric convolutions (1 × 11 and 11 × 1 kernels) are introduced to split the spatial feature extraction directions. This design reduces computational consumption while more efficiently capturing direction-specific features (e.g., long-range dependencies in horizontal or vertical directions). Without significantly increasing the number of parameters, it effectively expands the receptive field, especially in scenarios requiring the capture of long-range spatial relationships. This branch structure enhances feature extraction efficiency and enables collaborative modeling of local and global information. Directionally sensitive strip convolutions can more effectively capture the features of strip-shaped or directionally oriented targets, thereby strengthening multi-scale modeling capabilities. By combining horizontal and vertical convolutions, IDC expands the receptive field while reducing reliance on deep-layer stacking, demonstrating superior performance in complex texture characterization and background interference resistance.

In YOLO-IWAL, IDC-CDFS is used for channel decomposition and dynamic feature scaling, which address the inherent limitations of traditional convolutional networks in adaptive feature learning. Conventional convolutional networks adopt fixed-weight strategies for channel features, failing to dynamically adjust the importance of each channel according to specific task requirements. This inflexibility inevitably leads to suboptimal feature fusion, especially in scenarios with complex targets and variable backgrounds. To tackle this issue, the CDFS block implements a three-stage feature optimization mechanism: First, channel decomposition is achieved through 1 × 1 convolution, which compresses redundant feature dimensions to generate compact feature representations. This operation not only reduces computational overhead but also guides the network to learn robust cross-channel correlations by emphasizing the interdependencies between different feature channels. Subsequently, activation functions are introduced to decompose and enhance key semantic information. Specifically, the non-linear transformation capability of activation functions enables the module to highlight task-relevant feature channels (e.g., cow teat contour and structural features) while adaptively suppressing redundant channels that encode irrelevant background information (such as udder skin textures and environmental noise). Finally, the original low-level feature information is re-aggregated via residual connections. Distinctively, the CDFS block incorporates learnable parameters to dynamically adjust residual weights: these parameters strengthen the contribution of task-correlated channels to the final feature map and further suppress noise interference, thereby improving the effectiveness of feature aggregation in deep feature extraction scenarios. By integrating channel decomposition, adaptive feature enhancement, and dynamic residual adjustment, the IDC-CDFS module achieves a better balance between feature representation capability and model robustness, laying a solid foundation for accurate cow teat detection in complex milking environments.

#### 4.2.2. Wavelet Pooling in the Proposed Model

Conventional CNN downsampling reduces spatial resolution and computational cost but inevitably discards fine-grained features—critical for detecting small, orientation-variable targets like cow teats. Isotropic convolutions lack directional sensitivity, and strided convolution often causes aliasing due to neglect of the Nyquist sampling principle, degrading detection accuracy.

To address these issues, we replace traditional downsampling modules in YOLO with a Wavelet Pooling module [18], which integrates multi-band decomposition and downsampling into a single convolutional operation. Unlike computationally expensive recursive wavelet transforms, this module uses four predefined normalized Haar wavelet filters, $h_{\mathrm{LL}}$, $h_{\mathrm{LH}}$, $h_{\mathrm{HL}}$, and $h_{\mathrm{HH}}$. The filter $h_{\mathrm{LL}}$ captures low-frequency global structures, whereas $h_{\mathrm{LH}}$, $h_{\mathrm{HL}}$, and $h_{\mathrm{HH}}$ capture high-frequency horizontal, vertical, and diagonal edge/detail information, respectively. The coefficient 0.5 is obtained from the outer product of orthonormal one-dimensional Haar low-pass and high-pass filters. This normalization helps maintain a stable feature scale across different frequency subbands, and the resulting filters are defined as follows:

(1) $$\begin{array}{cc} h_{\mathrm{LL}} & =\left[\begin{array}{cc} 0.5 & 0.5\\ 0.5 & 0.5 \end{array}\right],\;h_{\mathrm{LH}}=\left[\begin{array}{cc} -0.5 & -0.5\\ 0.5 & 0.5 \end{array}\right],\\ h_{\mathrm{HL}} & =\left[\begin{array}{cc} -0.5 & 0.5\\ -0.5 & 0.5 \end{array}\right],\;h_{\mathrm{HH}}=\left[\begin{array}{cc} 0.5 & -0.5\\ -0.5 & 0.5 \end{array}\right] \end{array}$$

For an input feature map of shape [B,C,H,W] (where *B* denotes batch size, *C* is the number of input channels, and *H* and *W* denote the feature map height and width, respectively), we utilized the following:

1. Filter Expansion: The base filter bank with dimensions [4,1,2,2] is replicated *C* times along the channel dimension, resulting in an expanded filter bank of [4C,1,2,2]—allocating 4 dedicated wavelet filters per input channel.
2. Grouped Convolution: With grouping set to *C*, each input channel is convolved independently with its corresponding 4 filters. A stride of 2 achieves spatial downsampling, yielding an output feature map of shape [B,4C,H/2,W/2].
3. Efficient Fusion: Channel expansion to 4C integrates multi-frequency components, while spatial downsampling compresses redundant data without losing key structural/detail information—avoiding the high complexity of recursive wavelet transforms.

Wavelet Pooling adheres to the Nyquist sampling principle, effectively mitigating aliasing and feature distortion. Its inherent directional sensitivity better captures the slender, variable orientations of cow teats, enhancing robustness under challenging conditions. By balancing receptive field expansion, multi-scale feature representation, and computational efficiency, the module reduces parameters and FLOPs while preserving shallow low-frequency structures and deep edge/texture features.

In the YOLO-IWAL framework, Wavelet Pooling is integrated at the 3rd, 5th, and 7th downsampling stages of the backbone. This strategic placement ensures sufficient retention of structural and detail information even at low resolutions, improving detection precision and inference efficiency for teat targets.

#### 4.2.3. AC-FPN

**Figure 6:**
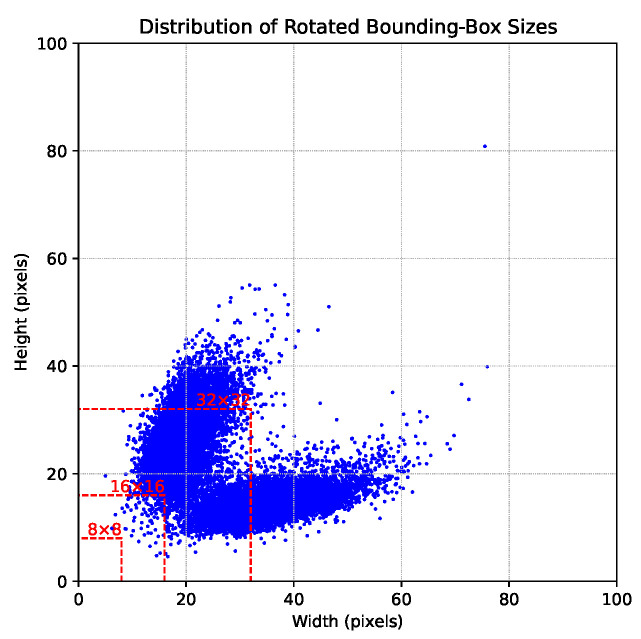
The distribution of bbox sizes of cow teats in the dataset.

The enhanced PAN structure in YOLO11 maintains bidirectional feature aggregation pathways in both top-down and bottom-up directions, incorporating the C3k2 module, C2PSA attention mechanism, and spatial pyramid pooling to improve multi-scale feature fusion efficiency and small-object detection performance. However, this architecture still exhibits certain feature redundancy, leading to increased parameter counts and computational demands. In the context of robotic milking, statistical analysis of bounding-box sizes in the self-constructed cow teat dataset indicates that very few teat instances require the high-resolution stride-8 P3 feature map. As shown in Figure 6, the distribution of bounding box sizes indicates that 23,581 detections fall between 16 × 16 and 32 × 32 in size, 104 detections are between 8 × 8 and 16 × 16, and 11,674 detections exceed 32 × 32. These results show that only 104 annotated instances, accounting for less than 0.3% of all annotations, fall within the 8×8 to 16×16 pixel range. Therefore, removing the stride-8 P3 feature map can reduce computational cost while having a limited impact on the detection of extremely small teats. Given this distribution, detection of extremely small objects may not be critical in milking-farm scenarios. Therefore, we propose removing the P3 feature layer to reduce model parameters and computational load while maintaining detection accuracy. Experimental validation confirms that this modification preserves performance metrics while significantly decreasing computational requirements.

Moreover, during the top-down propagation, high-level semantic features undergo multiple downsampling operations, which easily leads to the loss of fine-grained information and results in missed detections. Additionally, due to the limited input resolution, low-level features tend to introduce noise when propagated upward, adversely affecting the reliability of high-level feature representations.

To address these issues, we introduce High-level Screening-feature Fusion Pyramid (HSFPN) [19]. Unlike the traditional FPN, which relies on simple multi-layer feature fusion to obtain multi-scale representations, HSFPN adopts a “hierarchical screening–branch fusion” strategy. Specifically, it first filters features at different levels to suppress redundant or inefficient information, and then divides the filtered features into several sub-pyramids, each of which independently performs feature fusion and upsampling. Through this layer-wise screening and multi-branch fusion design, HSFPN preserves multi-scale representation capability while achieving more efficient cross-layer interaction, effectively reducing redundant computation and improving detection performance for objects with large scale variations.

Regarding traditional attention mechanisms, modules such as CBAM [20], ECA [21], and SE [22] have proven effective for enhancing feature representation, but they still exhibit certain limitations. The spatial attention in CBAM relies on fixed pooling strategies, which makes it difficult to adaptively adjust feature weights based on input content. ECA models only channel dependencies while ignoring spatial structural information, making it less effective when dealing with small targets or objects with large scale variations. Overall, traditional attention mechanisms lack sufficient flexibility to handle complex backgrounds and multi-scale target variations.

**Figure 7:**
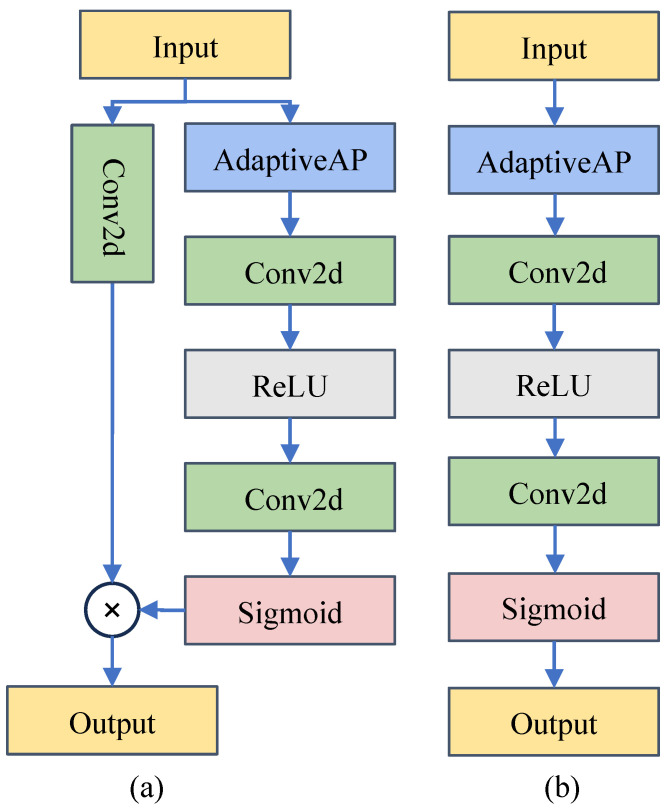
Attention Convolution Block. Subfigure (**a**) shows the AC Block for feature selection in the Neck of YOLO-IWAL; Subfigure (**b**) shows the AC Block for feature fusion.

To address the limitations of conventional convolutional operations, this paper proposes an Attention Convolution (AC) Block. The architectural design of the AC Block is illustrated in Figure 7. This module retains the local feature extraction capabilities of standard convolution while introducing a dynamic attention mechanism to enhance feature selection and fusion. The proposed AC module generates channel-wise attention weights through a lightweight attention generation network. These weights are conditioned on the input feature map, enabling adaptive regulation of channel responses according to the input features. Specifically, the input feature map undergoes adaptive global average pooling to aggregate global spatial information into a channel descriptor. Subsequently, two successive 1×1 convolutions are applied to reduce and restore the channel dimensions. Finally, a Sigmoid activation function produces channel-wise attention weights, which are broadcast across the spatial dimensions. The final output is computed as the element-wise multiplication of the convolutional output and the broadcast attention weights.

The corresponding calculation formula is as follows:

(2) conv_out=Conv(x)

(3) $$A\left(x\right)=\sigma \left({\mathrm{Conv}}_{2}\left(\mathrm{ReLU}\left({\mathrm{Conv}}_{1}\left(\mathrm{AdaptiveAvgPool}(x)\right)\right)\right)\right)$$

The final output is given by

(4) output=conv_out×A(x)

This dynamic weighting process enables adaptive regulation of channel responses according to the input features, allowing the network to emphasize informative feature channels while suppressing redundant responses. When integrated into the FPN structure, the proposed module can dynamically adjust feature responses during multi-scale feature fusion according to inter-layer differences and feature importance, thereby enhancing the representation of fine-grained details. In bovine udder images, where teat targets exhibit variations in scale and appearance, this design improves informative feature selection and contributes to more robust teat detection under complex imaging conditions.

#### 4.2.4. OBB_LSDCD

The original YOLO11 detection head adopts depthwise separable convolutions to reduce computational cost; however, its ability to capture high-frequency edges and directional details is limited, which hampers performance when detecting oriented objects. Meanwhile, Batch Normalization tends to exhibit unstable statistics under small batch sizes, whereas Group Normalization (GN) has been shown to offer more stable training dynamics and better generalization in detection tasks [23].

**Figure 8:**
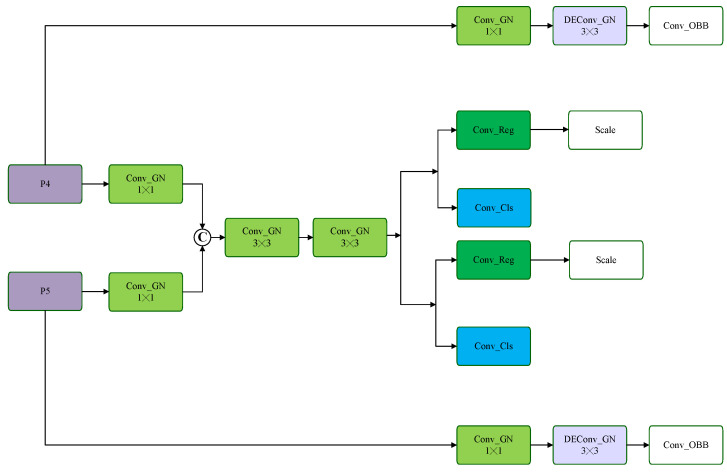
Architecture of the OBB_LSDCD module. GN denotes group normalization.

To enhance the feature representation capability and inference efficiency of YOLO11 in oriented bounding box detection tasks, we propose the Lightweight Shared Detail-enhanced Convolution Detector (LSDCD). The overall network architecture integrating the proposed LSDCD module is illustrated in Figure 8, where LSDCD is embedded into the detection head of YOLO11 to replace the original depthwise separable convolution layer, thereby strengthening the model’s ability to handle oriented targets. Considering that teat regions often exhibit weak edges and blurred textures, relying solely on standard convolutions is insufficient for recovering clear and discriminative contours. Therefore, we introduce detail-enhanced convolution (DEConv) [24] with edge-aware priors to enhance gradient-sensitive feature extraction. This module integrates GN, group convolution, and DEConv to strengthen the network’s ability to perceive high-frequency gradient information and fine-grained structural details. It is particularly effective for detecting OBB targets such as teats in low-resolution and low-contrast bovine udder images.

**Figure 9:**
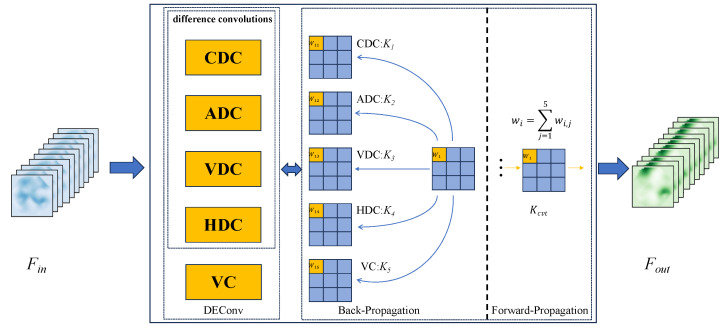
Architecture and re-parameterization procedure of the DEConv module. VC, CDC, HDC, VDC, and ADC denote vanilla, central difference, horizontal difference, vertical difference, and angular difference convolutions, respectively.

The network structure diagram of the DEConv module and the reparameterization process during training and inference are shown in Figure 9. As can be observed from the figure, the DEConv module adopts a multi-branch structure consisting of a standard convolution branch (VC) and four differential convolution branches—central difference convolution (CDC), horizontal difference convolution (HDC), vertical difference convolution (VDC), and angular difference convolution (ADC). CDC captures local gradients through central differences; HDC and VDC extract gradient variations along the horizontal and vertical directions, respectively; and ADC aggregates directional discrepancies from multiple orientations to better encode rotated boundaries. The outputs of all branches are fused via element-wise addition, producing composite features that simultaneously retain intensity information and high-frequency gradient cues. This design enables more accurate localization of teat edges and orientation details under complex backgrounds and low-contrast imaging conditions.

During inference, DEConv employs reparameterization to compress its multi-branch structure into a single equivalent convolution kernel, enabling the same computational cost as a standard convolution. This mechanism preserves gradient sensitivity while significantly improving inference efficiency, making LSDCD both accurate and deployment-friendly for small-scale oriented object detection.

### 4.3. Siamese Network-Based Tracking Algorithm

#### 4.3.1. Visual Encoding Backbone

Considering the limited computing resources of automatic milking robot terminals and the requirement for real-time performance, the feature extraction backbone needs to achieve a balance between lightweight design and feature representation capability. The visual encoding backbone of the Siamese network adopts MobileNetV3-Small. This network is based on depthwise separable convolutions, which significantly reduce the number of parameters and computational cost while maintaining strong feature extraction capability, making it suitable for resource-constrained embedded deployment scenarios. During training, ImageNet pre-trained weights are loaded for initialization, allowing the low-level texture and mid-level structural features learned from large-scale natural images to accelerate convergence. Meanwhile, the backbone is fine-tuned with a relatively low learning rate to avoid disrupting the existing feature representation capability.

#### 4.3.2. Fourier Positional Encoder

**Figure 10:**
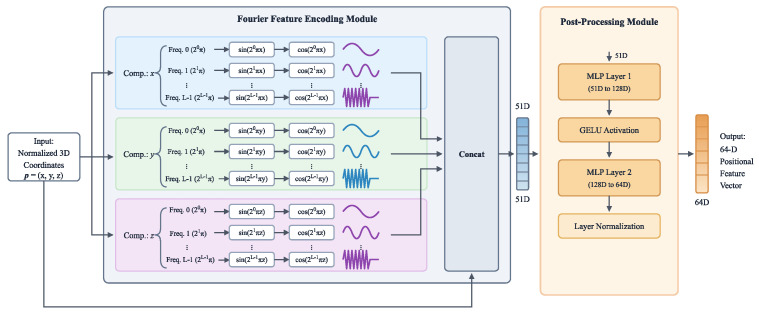
Architecture of the Fourier Positional Encoder.

Neural networks generally exhibit spectral bias, tending to learn low-frequency and smooth features while having difficulty capturing high-frequency details. Three-dimensional coordinates are inherently low-dimensional physical signals with relatively low-frequency characteristics. If raw 3D spatial coordinates are directly concatenated with high-dimensional visual features extracted by a deep network, the spatial geometric information can easily be overwhelmed by the dominant visual gradients during backpropagation, making subtle numerical differences difficult for the network to utilize effectively. To bridge this gap in dimensionality and representation capacity, this study adopts Fourier positional encoding to expand the 3D coordinates into a high-dimensional representation. This method uses sine and cosine basis functions at different frequencies to map low-dimensional continuous coordinates into high-dimensional feature vectors rich in frequency information, thereby balancing the representation capacity of spatial geometric information and visual appearance information. Specifically, as shown in Figure 10, each scalar component *p* of the normalized 3D coordinate p is mapped using *L* groups of basis functions, and the encoding formula is defined as follows:

(5) $$\gamma \left(p\right)=\left[p,\sin\left(2^{0}\cdot \pi p\right),\cos\left(2^{0}\cdot \pi p\right),\ldots ,\sin\left(2^{L-1}\cdot \pi p\right),\cos\left(2^{L-1}\cdot \pi p\right)\right]$$

where *p* denotes a scalar component of the normalized 3D coordinate p, and *L* denotes the number of frequency levels and is set to 8 in this study. In this multi-scale representation, the low-frequency components capture the global absolute position and spatial distribution trend of the target, while the high-frequency components amplify subtle variations in the coordinate space, enabling the network to remain sensitive to fine spatial displacement. This mechanism allows teats that are spatially close but not identical to obtain highly discriminative encoded representations. Finally, the encoded high-frequency and high-dimensional vector is mapped by a two-layer perceptron and layer normalization into a 64-dimensional positional feature. This not only effectively matches the dimensionality of the visual features, but also provides a solid mathematical basis for adaptive feature weighting in the subsequent fusion mechanism.

#### 4.3.3. Adaptive Geometric Encoder

**Figure 11:**
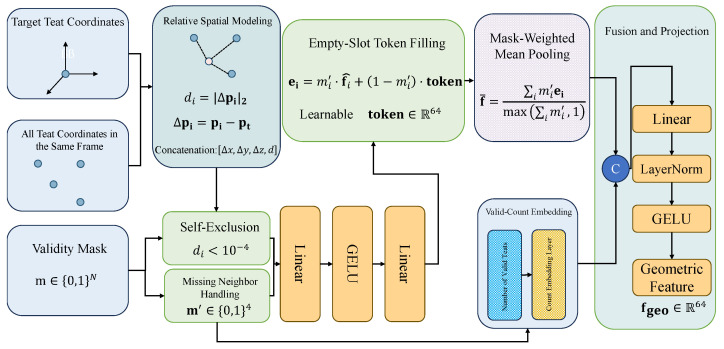
Network Architecture of Adaptive Geometric Encoding.

The absolute coordinate of a single cow teat may undergo global translation or rotation due to changes in cow posture. Therefore, relying only on the positional encoding of an individual teat is insufficient to provide stable identity cues. Since cows typically have four teats, their relative spatial arrangement contains strong physiological structural priors. In this study, an adaptive geometric encoder is designed to model the relative spatial relationships between the target teat and the other teats in the same frame. The network structure of this module is shown in Figure 11.

This module takes the target teat coordinate, the coordinates of all teats in the same frame, and a validity mask as inputs. For each neighboring teat, the 3D displacement vector Δp and Euclidean distance *d* between the neighbor and the target teat are calculated and concatenated into a four-dimensional relative feature [Δx,Δy,Δz,d], which is then encoded into a neighbor embedding by a multi-layer perceptron. To exclude the target teat itself from the neighboring context, if the Euclidean distance between a teat in the same frame and the target teat satisfies $d<1\times 10^{-4}$, that teat is identified as the target itself and is not used as a neighbor in the subsequent geometric feature encoding. Here, *d* is calculated from Z-score-normalized 3D coordinates; therefore, the threshold $1\times 10^{-4}$ is dimensionless. The validity mask is used to handle missing neighboring teats caused by occlusion or missed detection. When the position of a neighboring teat is invalid, a learnable empty-neighbor token is used for padding to avoid interference from zero values during aggregation. The encoded features of all valid neighbors are aggregated into a geometric context vector through mask-weighted mean pooling. Meanwhile, a count embedding layer is introduced to encode the number of valid teats detected in the current frame as an additional feature. This feature is concatenated with the geometric context and then passed through a projection layer to output a 64-dimensional geometric feature. This design enables the network to perceive the local spatial configuration of teats. Even when the target undergoes global translation, its relative arrangement with neighboring teats remains stable, thereby providing identity-discriminative information complementary to absolute position.

#### 4.3.4. Gated Fusion Mechanism

Visual features and geometric features characterize teat identity from the appearance and spatial dimensions, respectively, and their reliability varies with scene conditions. When occlusion is limited, visual features usually have stronger discriminative ability; when teat appearances are similar or partial occlusion occurs, geometric features can provide critical distinguishing cues. Simple feature concatenation or addition cannot adaptively adjust the contribution of the two modalities according to specific conditions.

To address this problem, this study adopts a gated fusion mechanism. First, the absolute positional feature and the relative geometric feature are concatenated and compressed through a linear layer to form an integrated spatial feature $f_{\mathrm{spatial}}$. Then, the visual feature $f_{\mathrm{vis}}$ and the integrated spatial feature are concatenated and fed into a gating network, where a Sigmoid activation function generates an element-wise gating weight vector $g\in {\left[0,1\right]}^{d}$. After the spatial feature is linearly projected to align with the dimensional space of the visual feature, it is multiplied by the gating weight element by element and then added to the visual feature. The fusion formula is defined as follows:

(6) $$f_{\mathrm{fused}}=f_{\mathrm{vis}}+g⊙W_{s}f_{\mathrm{spatial}}$$

where $W_{s}$ is the projection matrix for the spatial feature, and ⊙ denotes element-wise multiplication. The fused result is passed through linear projection and layer normalization to produce a 128-dimensional output, which is finally projected onto the unit hypersphere through L2 normalization. During training, the gating network automatically learns the degree of trust assigned to the two modalities under different conditions, without requiring a manually predefined fusion ratio.

The basic backbone adopts the lightweight visual backbone MobileNetV3-Small, while a complete spatial encoding branch is added after the embedding layer. The Fourier positional encoder maps 3D coordinates into multi-frequency positional representations, enhancing fine-grained spatial resolution. The adaptive geometric encoder takes the relative displacement and distance between the target teat and its neighboring teats in the same frame as inputs, while introducing empty-neighbor tokens and valid target counts to address feature drift caused by fluctuations in the number of visible points. The gated fusion mechanism adaptively adjusts the injection strength of spatial information, preventing geometric noise from directly overriding visual semantics. Conventional 2D Siamese networks usually perform only late-stage feature concatenation or distance calculation, whereas this study introduces learnable fusion in the forward stage. As a result, the representation sources are richer, the fusion pathway is more refined, and the final normalized identity vectors become more discriminative for cross-frame matching.

#### 4.3.5. Association Cost Matrix and Hungarian Matching

During inference, the Siamese network generates a normalized identity vector for each detected teat in two adjacent frames. Pairwise cosine distances between the identity vectors from the previous and current frames are then calculated to construct an association cost matrix. The Hungarian algorithm is applied to the cost matrix to obtain the minimum-cost inter-frame assignment. Since the algorithm supports rectangular cost matrices, it can handle variations in the number of visible teats between adjacent frames. Finally, assigned pairs with costs exceeding a predefined threshold are rejected as unreliable matches.

## 5. Experiments

### 5.1. Experiment Setting

The experiments include model training, performance evaluation, and edge deployment for both the detection model and the Siamese network-based tracking algorithm. For the detection model, YOLO11n-OBB was adopted as the baseline network. The number of training epochs was set to 200, with a batch size of 32. Automatic Mixed Precision (AMP) was enabled to accelerate training and reduce memory consumption. Stochastic Gradient Descent (SGD) was used as the optimizer, with an initial learning rate of $1\times 10^{-2}$ and a momentum of 0.9. The default online data augmentation strategies in YOLO11, including Mosaic augmentation, HSV color augmentation, geometric transformation, and horizontal flipping, were used to improve model generalization.

For the tracking model, AdamW was used as the optimizer to improve model convergence. The initial learning rate was set to $1\times 10^{-4}$; the batch size was set to 32, and the model was trained for 60 epochs.

The hardware and software configurations of the experiments are summarized in Table 1. For edge deployment, the Rockchip RK3588 development board was used as the hardware platform, with an NPU computing capability of 6 TOPS. The NPU driver version was 0.9.6, and the Rockchip Neural Network Runtime Library (librknnrt) version was 2.3.0.

### 5.2. Evaluation Metrics

To comprehensively evaluate the proposed detection model, widely used object detection metrics were adopted, including Precision, Recall, mean Average Precision (mAP), number of parameters, computational complexity (FLOPs), inference speed, and model size.

Precision quantifies the proportion of correctly predicted positives among all predicted positives, while Recall measures the proportion of actual positives that are successfully detected:

(7) $$P=\frac{\mathrm{TP}}{\mathrm{TP}+\mathrm{FP}}$$

(8) $$R=\frac{\mathrm{TP}}{\mathrm{TP}+\mathrm{FN}}$$

where TP, FP, and FN denote true positives, false positives, and false negatives, respectively. A higher Precision indicates fewer false positives, while a higher Recall indicates fewer missed detections.

AP is defined as the area under the Precision–Recall (P–R) curve for a single category:

(9) $$\mathrm{AP}=\int_{0}^{1}P\left(R\right)\;dR$$

where P(R) denotes Precision at a given Recall *R*. For multi-class tasks, the mean Average Precision is defined as:

(10) $$\mathrm{mAP}=\frac{1}{C}\sum_{c=1}^{C}{\mathrm{AP}}_{c}$$

where *C* is the total number of categories and ${\mathrm{AP}}_{c}$ is the Average Precision for class *c*. In practice, mAP is often reported under different IoU thresholds. **mAP50** is calculated at a single IoU threshold of 0.5, while **mAP50-95** is averaged over 10 IoU thresholds from 0.5 to 0.95 with a step size of 0.05, providing a stricter evaluation of localization quality. Since this study focuses on a single class (cow teats), mAP reduces to the AP of this class, and mAP50 is used as the primary metric for evaluating detection performance.

To evaluate the data association ability of the tracking algorithms independently from detector performance, the tracking comparison was conducted using manually annotated boxes as the common input for all compared trackers. Under this setting, false positives and false negatives caused by detection are fixed or excluded, making MOTA less informative for comparing inter-frame association performance. Therefore, this study mainly evaluates the tracking algorithm from the perspective of identity association.

During final evaluation, all teat features from each pair of adjacent frames in the test set are extracted to construct a complete cost matrix, and the Hungarian algorithm is used to obtain the global optimal matching. The matching accuracy, denoted as ACC, is defined as follows:

(11) $$\mathrm{ACC}=\frac{\sum_{k=1}^{K}I\left({\mathrm{ID}}_{\mathrm{pred}}^{k}={\mathrm{ID}}_{\mathrm{gt}}^{k}\right)}{K}$$

where *K* denotes the total number of evaluated teat association pairs, and I is the indicator function, which equals 1 when the predicted identity ID matches the ground-truth ID and 0 otherwise.

To evaluate identity preservation over complete video sequences, this study additionally adopts the standard identity F1 score (IDF1), which is defined as:

(12) $$\mathrm{IDF}1=\frac{2\mathrm{IDTP}}{2\mathrm{IDTP}+\mathrm{IDFP}+\mathrm{IDFN}}$$

where IDTP, IDFP, and IDFN denote the numbers of correctly identified, incorrectly identified, and missed identity detections, respectively, obtained by globally matching predicted and ground-truth trajectories over the complete test clips. A higher IDF1 indicates more reliable identity preservation throughout the evaluated video sequences.

### 5.3. Ablation Study

#### 5.3.1. AC-FPN Ablation Study

To evaluate the effectiveness of AC-FPN, it was compared with representative attention-based FPN variants, including SEAttention-FPN, ECA-FPN, ELA-FPN [25], and SimAM-FPN [26]. As shown in Table 2, AC-FPN achieves the highest precision of 99.69% and the highest mAP50-95 of 95.75%, while maintaining an inference speed of 1556.9 FPS. Although ECA-FPN obtains a higher recall and SEAttention-FPN achieves a faster inference speed, AC-FPN provides a more favorable balance between detection accuracy, localization quality, and computational efficiency.

**Figure 12:**
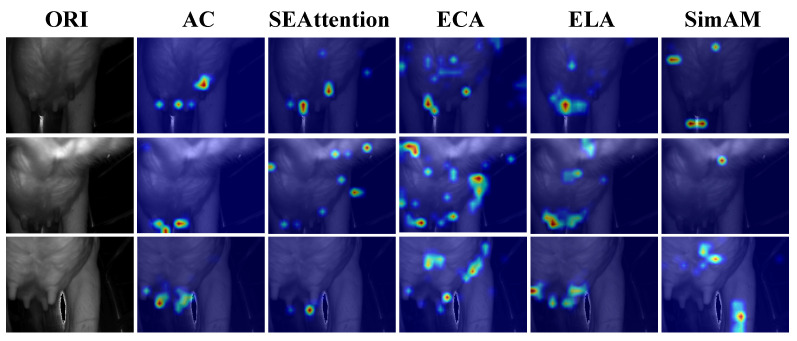
Comparison of Feature Map Effects with Various Attention Mechanisms.

GradCAM [27] heatmaps are further used to qualitatively compare the feature localization capabilities of different FPN variants, as shown in Figure 12. AC-FPN consistently concentrates its responses on cow teat regions across different scenes, whereas several comparison methods exhibit weaker target responses or additional attention to background interference. These visualization results further support the effectiveness of AC-FPN in selecting and fusing informative teat features.

#### 5.3.2. YOLO-IWAL Ablation Study

The ablation experiment results in Table 3 show how each YOLO-IWAL modification contributes to the detector. Taking the baseline model (Method 1) as the reference, the individual introduction of IDC-CDFS, Wavelet Pooling, AC-FPN, or LSDCD produces different but complementary effects while maintaining the stability of mAP50 around 99.49%. IDC-CDFS reduces parameters and FLOPs by improving backbone feature extraction efficiency, Wavelet Pooling lowers the computational load while preserving fine structural details during downsampling, AC-FPN increases inference speed by reducing redundant neck computation after feature screening and fusion, and LSDCD improves detection precision by strengthening orientation-sensitive boundary representation in the OBB head.

The performance is further synergistically optimized after combining multiple modules: the fusion of IDC-CDFS, Wavelet Pooling, and AC-FPN (Method 7) reduces the number of parameters to 1.96M and the computational load to 3.4G, while maintaining high-speed inference of 1550.8 FPS; the final YOLO-IWAL integrating all four task-matched modifications (Method 8) achieves the optimal comprehensive performance, with a precision of 99.69%, only 1.85M parameters, 3.4G computational load, and an inference speed of 1556.9 FPS. These results support the design rationale that detector efficiency is mainly gained by pruning and simplifying feature paths according to the teat-scale distribution, while detection robustness is preserved by detail-aware backbone and OBB-head modifications.

### 5.4. Ablation Study of the Siamese Tracking Algorithm

To verify the performance of the core modules in the Siamese network-based tracking algorithm, a progressive ablation study was designed under the same hardware and software testing environment. A pure 2D visual Siamese network containing only the MobileNetV3-Small visual backbone was used as the baseline model. On this basis, the Fourier positional encoder, adaptive geometric encoder, and gated spatial fusion mechanism were gradually introduced. The results of the core module ablation study are shown in Table 4.

The experimental results show that the pure visual baseline, Model A, performs poorly when tracking highly homogeneous teat targets. After introducing the Fourier positional encoder, Model B gains absolute spatial perception capability, and ACC increases by 8.26 percentage points, confirming the effectiveness of high-frequency mapping of physical coordinates for fine-grained identity discrimination. Based on Model B, the adaptive geometric encoder is further introduced to construct Model C, enabling the network to model local spatial topology. Even when a teat is temporarily occluded, the model can rely on the relative spatial configuration of the remaining visible teats to maintain identity association, improving both ACC and IDF1. Model D replaces simple channel concatenation with a gated spatial fusion mechanism. When visual features are affected by illumination variation or motion blur, the gated mechanism adaptively balances visual and geometric information by increasing the contribution of geometric features. As a result, Model D achieves the best performance among the evaluated configurations, with ACC reaching 74.59% and IDF1 reaching 93.24%. The difference between ACC and IDF1 reflects their complementary evaluation scopes: ACC evaluates exact identity associations between individual adjacent frames and remains sensitive to low-frame-rate and abrupt-motion conditions, whereas the higher IDF1 indicates that the proposed method preserves overall teat identity consistency across complete video sequences. Accordingly, a limited number of local association errors can reduce ACC without necessarily producing persistent identity mismatches across an entire trajectory, and may therefore have a smaller effect on IDF1. These results suggest that, although exact adjacent-frame association remains challenging in some cases, the proposed tracking architecture improves sequence-level identity preservation in complex dynamic milking scenarios.

### 5.5. Comparative Analysis

#### 5.5.1. Detection Algorithm Comparison

To validate the performance of the improved YOLO model on the dairy cow teat detection task, this study conducts a comparative experiment against state-of-the-art networks in the field of rotated object detection. Under identical experimental conditions, we apply 9 rotated-object-detection algorithms—Rotated FasterRCNN [28], S2A Net [29], Redet [30], R3det [31], Roi Trans [32], Rotated Retinanet [33], Kld [34], GWD [35], and Kfiou [36]—to the same dataset and perform detection analysis. Among them, three models, Kld, GWD, and Kfiou, used the same training network (rotated_retinanet); only different loss functions were employed during training. The comparison also includes representative YOLO models, including YOLOv8n, YOLO11n, YOLO11s, YOLO12n, and YOLO13n. Table 5 presents a quantitative comparison between the proposed YOLO-IWAL and 14 comparison detectors. In terms of accuracy, YOLO-IWAL attains the highest Precision of **99.69%** and a competitive mAP50 of **99.49%**. While traditional models like R3Det and RoI Transformer yield respectable accuracy, they incur heavy computational overheads, with parameters exceeding 40 M and GFLOPs ranging from 30 to 46 G. In contrast, the proposed YOLO-IWAL significantly optimizes the model complexity, requiring only **1.85 M** parameters and **3.4 GFLOPs**, which is the lowest among all tested models.

Most notably, the inference speed of YOLO-IWAL reaches **1556.9 FPS**, representing a substantial improvement over the standard YOLOv8n (1031 FPS) and being approximately 14.6 times faster than the Rotated Faster R-CNN (106.5 FPS). These results demonstrate that YOLO-IWAL successfully eliminates redundant computations, offering a robust and efficient solution for real-time dairy cow teat detection in resource-constrained environments.

#### 5.5.2. Detection Performance Across Different Test Subsets

To further assess the robustness of YOLO-IWAL under different test conditions, the detection test set was divided into several diagnostic subsets based on the ground-truth OBB annotations, manual review, and acquisition timestamps. The inclined-teat subset contains 488 images in which at least one teat OBB has a long-axis deviation from the vertical direction of no less than 30°. In addition, a partial-occlusion subset containing 654 images was constructed through manual review. An image was included when at least one visible teat was judged to be visibly incomplete because of occlusion. Furthermore, all test images were partitioned into three acquisition groups according to their original acquisition timestamps, containing 459, 721, and 627 images, respectively. These acquisition groups were used to assess the consistency of the detector across different data-collection sessions.

The results are presented in Table 6. YOLO-IWAL achieved a Precision of 99.69%, a Recall of 99.01%, and an mAP@50 of 99.49% on the complete test set. On the inclined-teat subset, the model achieved a Precision of 99.15%, a Recall of 98.15%, and an mAP@50 of 99.37%. The partial-occlusion subset achieved a Precision of 99.75%, a Recall of 97.59%, and an mAP@50 of 99.32%. These results show that teat inclination and partial occlusion increase the difficulty of detection, particularly in terms of Recall; nevertheless, the detector maintained an mAP@50 above 99.3% on both challenging subsets. Across the three groups collected at different acquisition times, Precision remained between 99.57% and 99.89%, Recall ranged from 98.58% to 99.17%, and mAP@50 remained between 99.49% and 99.50%. This indicates consistent detection performance across the evaluated data-collection sessions.

#### 5.5.3. Tracking Algorithm Comparison

**Figure 13:**
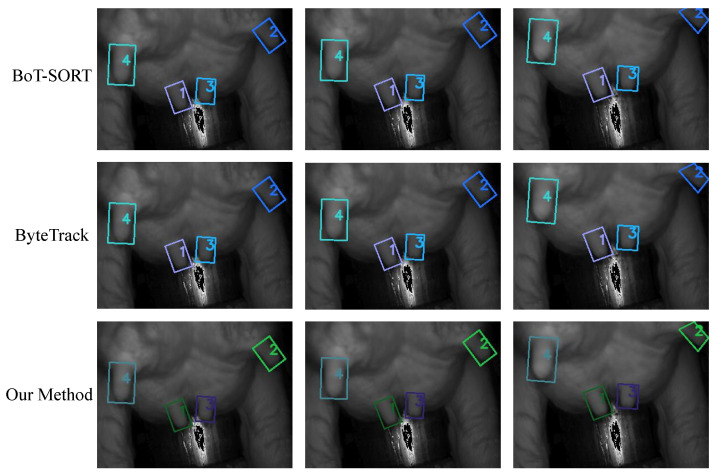
Visualization Diagram of Tracking Performance for Different Algorithms Under Stable Bovine Conditions.

Figure 13 presents the qualitative evaluation results of different tracking algorithms under practical automatic milking conditions. In Figure 13, when the cow remains relatively stationary, the spatial displacement of the teats is small, and all compared algorithms maintain stable tracking without identity loss or incorrect association.

**Figure 14:**
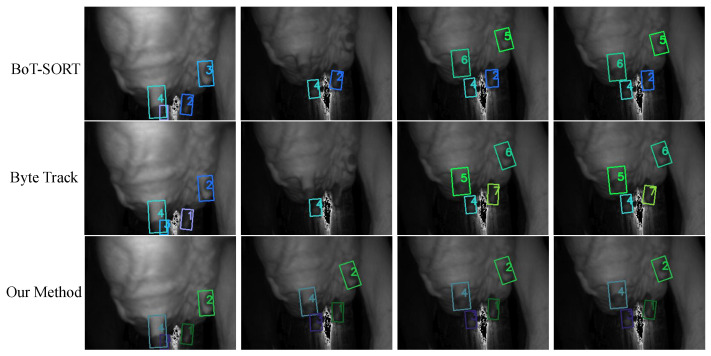
Visualization Diagram of Tracking Performance for Different Algorithms Under Violent Cow Shaking. The numbers shown in the figure are tracker-assigned trajectory labels rather than the anatomical teat identity labels defined in the dataset.

However, substantial cow movement is unavoidable during practical milking operations. Under the abrupt motion condition shown in Figure 14, BoT-SORT and ByteTrack exhibit limited robustness against rapid and nonlinear teat movement from the second frame onward. By the third frame, despite the continuous provision of manually annotated ground-truth bounding boxes, both algorithms fail to preserve the identities of teats 1–3. Moreover, the remaining track 4 is incorrectly associated with a teat different from the target identified in the first frame. In contrast, the proposed method consistently maintains the identities of all teats throughout the sequence, illustrating its ability to preserve teat identities in this representative abrupt-motion sequence.

As shown by the quantitative results in Table 7, conventional multi-object tracking algorithms exhibit varying limitations under the low-frame-rate sampling and abrupt motion conditions contained in the tracking dataset. In contrast, the proposed method achieves the best identity association performance among the evaluated methods, reaching an ACC of 74.59% and an IDF1 of 93.24%. Although its inference speed is lower than those of the compared methods, it still reaches 1328 FPS on the NVIDIA RTX 3080Ti platform, sufficiently satisfying the real-time tracking requirements of the system. These results demonstrate that the proposed method provides more reliable teat identity association under low-frame-rate imaging and abrupt cow movement while maintaining sufficient inference speed for real-time tracking.

#### 5.5.4. Displacement-Stratified Evaluation of the Proposed Tracking Method

To evaluate the influence of large inter-frame displacement on the proposed tracking method, displacement-stratified experiments were conducted on the tracking test set. For each adjacent-frame pair, the Euclidean displacement of each teat that remained visible in both frames was calculated from its stored 3D endpoint coordinates as $d_{t,i}={∥p_{t+1,i}-p_{t,i}∥}_{2}$, where $p_{t,i}$ denotes the 3D endpoint coordinate of teat *i* at frame *t*. The mean displacement of all persistent teat associations within each video clip was then used as the clip-level displacement measure. To avoid using test-set information during subset construction, the threshold was determined from the training set only. Specifically, the 75th percentile of the training clip-level displacement distribution was selected, yielding τ=45.52 in the stored 3D coordinate units. Test clips with a mean displacement greater than or equal to τ were assigned to the high-displacement subset, whereas the remaining clips formed the lower-displacement subset.

As shown in Table 8, the proposed tracking method achieves an ACC of 74.59% and an IDF1 of 93.24% on the overall test set. On the high-displacement subset, the ACC and IDF1 are 72.76% and 90.20%, respectively. Compared with the overall test-set results, the performance decreases by only 1.83 percentage points in ACC and 3.04 percentage points in IDF1 despite the larger inter-frame displacement. On the lower-displacement subset, the proposed method achieves an ACC of 79.93% and an IDF1 of 95.06%, both higher than the overall test-set results. These results demonstrate stable identity-association performance across different inter-frame displacement conditions, with the method maintaining an IDF1 of 90.20% despite a decrease in exact adjacent-frame association accuracy under large-displacement conditions.

### 5.6. Edge Deployment and Performance Analysis

#### 5.6.1. Deployment of YOLO-IWAL on RK3588

To verify the practical applicability of YOLO-IWAL in edge computing scenarios, we deployed the model on the Rockchip RK3588 AI development board and conducted performance testing on the test set of the self-constructed dataset (configuration: nmsThresh = 0.6, objectThresh = 0.5). For efficient edge deployment, post-training INT8 quantization was performed in RKNN Toolkit2. A total of 128 ToF amplitude images from the detection training set were resized to 640×640 pixels and used for calibration. Three calibration algorithms, namely normal, MMSE, and KL-divergence, were evaluated together with layer-wise and channel-wise quantization methods. Experimental results in Table 9 show that the quantized YOLO-IWAL variant achieves the highest precision and mAP50 among the evaluated configurations while maintaining competitive recall. Although its inference latency is higher than that of the official YOLO11n-OBB model due to differences in structural and operator design, the removal of one detection head reduces the post-processing time by 52.8% compared to the quantized version of the original model. Finally, in the single-core NPU operating mode, the proposed YOLO-IWAL model achieves an average inference speed of 22.9 FPS while maintaining a smaller weight memory footprint than the original baseline model. This inference speed is higher than the practical ToF acquisition rate of approximately 8 FPS under the tested milking-scene imaging configuration, demonstrating its suitability for real-time teat detection on resource-constrained edge hardware.

#### 5.6.2. Deployment of the Siamese Network on RK3588

To verify the real-time tracking capability of the Siamese network in edge computing scenarios, inference performance tests using FP16, INT8, and UINT8 precision formats were conducted on the RK3588 platform. As shown in Table 10, the INT8-quantized model achieves the highest full-pipeline processing speed of 166.39 FPS, including preprocessing, inference, postprocessing, and pair matching. This processing speed is substantially higher than the practical ToF acquisition rate of approximately 8 FPS under the tested milking-scene imaging configuration, providing sufficient computational margin for real-time teat identity association.

## 6. Conclusions

This study presents a cow teat detection and tracking framework comprising the YOLO-IWAL detector and a Siamese network-based tracking algorithm for automatic milking robots. YOLO-IWAL improves YOLO11n-OBB through IDC-CDFS, Wavelet Pooling, AC-FPN, and OBB_LSDCD, while maintaining detection accuracy and reducing redundant computation. The tracking algorithm combines visual appearance features with 3D spatial information through Fourier positional encoding, adaptive geometric encoding, and gated fusion. The generated identity vectors are used to construct an association cost matrix, and the Hungarian algorithm is applied to achieve inter-frame teat identity association.

Experimental results independently demonstrate the effectiveness of the proposed detection and tracking methods. YOLO-IWAL achieves a precision of 99.69% and an mAP50 of 99.49% while reducing the model complexity to 1.85M parameters and 3.4 GFLOPs and reaching an inference speed of 1556.9 FPS. The proposed tracking algorithm achieves an ACC of 74.59% and an IDF1 of 93.24% under low-frame-rate and abrupt-motion conditions. On the RK3588 platform, YOLO-IWAL reaches 22.9 FPS, while the INT8-quantized Siamese network achieves a tracking processing speed of 166.39 FPS. These results demonstrate that both methods provide reliable performance and satisfy their respective real-time requirements on resource-constrained edge hardware.

Despite the demonstrated detection, tracking, and edge-deployment performance, several limitations remain. The datasets were collected from a single commercial dairy farm, and further validation across different farms, cow breeds, and operating conditions is required. In addition, the detector and tracking modules were deployed and evaluated separately; end-to-end validation from online ToF acquisition and 3D endpoint recovery to robot-side coordinate output remains to be conducted. Future work will integrate the perception modules with the robotic platform and evaluate the complete closed-loop cup-attachment process. Moreover, the current framework focuses on teat position detection and identity tracking without explicitly estimating the complete spatial geometry and orientation of individual teats. Future work will combine SAM-assisted 3D point-cloud segmentation with PCA [37] or learning-based 6D pose estimation to obtain comprehensive teat pose information and support pose-adaptive cup attachment. 

## Figures and Tables

**Table 1:** Environmental configuration.

	Configuration Item	Value
Hardware	CPU	Ryzen™ 7 5800X
	RAM	16 GB
	GPU	NVIDIA RTX 3080 Ti
Software	Operating System	Ubuntu 22.04.5 LTS
	Deep Learning Framework	PyTorch 2.2.2
	CUDA Version	12.1
	Python Version	3.10.12

**Table 2:** Performance of the FPN part with different categories of attention mechanisms.

Model	P (%)	R (%)	mAP50 (%)	mAP50-95 (%)	FPS (Frame/s)
AC-FPN	99.69	99.01	99.49	95.75	1556.9
SEAttention-FPN	99.52	98.89	99.49	95.52	1687.5
ECA-FPN	99.28	99.52	99.48	95.32	1683.4
ELA-FPN	99.61	99.01	99.49	95.66	1590.5
SimAM-FPN	99.55	99.01	99.49	95.31	1610.3

**Table 3:** Ablation studies for each module on a self-constructed dataset.

Methods	IDC -CDFS	Wavelet Pooling	AC -FPN	LS DCD	P (%)	R (%)	mAP50 (%)	Params (M)	GFLOPs (G)	Size (M)	FPS (Frame/s)
1. BASE					99.49	99.21	99.49	2.65	6.6	5.5	1039.2
2	√				99.61	99.02	99.49	2.46	6.1	5.1	1050.9
3		√			99.64	99.13	99.50	2.30	5.7	4.8	1033.8
4			√		99.66	99.02	99.49	2.50	4.7	5.0	1565.7
5				√	99.54	99.20	99.49	2.27	6.1	5.1	1076.4
6	√	√			99.53	99.02	99.49	2.11	5.3	4.4	1037.0
7	√	√	√		99.59	99.04	99.49	1.96	3.4	4.0	1550.8
8	√	√	√	√	99.69	99.01	99.49	1.85	3.4	4.2	1556.9

Note: √ indicates that the corresponding module is enabled.

**Table 4:** Ablation study results of core tracking modules.

Model ID	Fourier Positional Encoder	Adaptive Geometric Encoder	Gated Spatial Fusion	ACC (%)	IDF1 (%)
Model A (Baseline)				63.81	86.41
Model B	✓			72.07	92.27
Model C	✓	✓		73.51	93.19
Model D (Ours)	✓	✓	✓	74.59	93.24

Note: ✓ indicates that the corresponding module is enabled. Model B and Model C use direct channel concatenation for feature fusion.

**Table 5:** Comparative experimental results for commonly used oriented bounding box object detection models.

Models	P (%)	mAP50 (%)	Params (M)	GFLOPs (G)	FPS (Frame/s)
kfiou	97.75	97.42	36.13	20.33	86.8
gwd	97.10	97.55	36.13	20.33	90.3
kld	97.52	97.29	36.13	20.33	87.6
r3det	97.26	98.12	41.58	31.89	61.8
redet	97.55	95.41	31.54	32.87	61.3
roi_trans	98.68	97.88	55.03	46.89	97.7
rotated_faster_rcnn	98.16	97.70	41.12	32.99	106.5
rotated_retinanet	98.17	97.15	36.13	20.33	87.0
s2anet	96.92	98.15	38.57	19.10	67.3
YOLOV8n	99.56	99.49	2.30	5.90	1031.0
YOLO11n	99.49	99.49	2.65	6.60	1039.2
YOLO11s	99.58	99.49	9.70	22.30	511.5
YOLO12n	99.59	99.49	2.66	6.70	712.8
YOLO13n	99.54	99.49	2.52	6.40	471.2
YOLO-IWAL	99.69	99.49	1.85	3.40	1556.9

**Table 6:** Detection performance of YOLO-IWAL on different test subsets.

Test Subset	Images	P (%)	R (%)	mAP50 (%)
Overall Test Set	1807	99.69	99.01	99.49
Inclined Teats (≥30°)	488	99.15	98.15	99.37
Partial Occlusion Subset	654	99.75	97.59	99.32
Acquisition Group 1	459	99.89	98.58	99.49
Acquisition Group 2	721	99.57	99.05	99.49
Acquisition Group 3	627	99.79	99.17	99.50

**Table 7:** Performance comparison of different tracking algorithms on the custom tracking dataset.

Tracking Algorithm	ACC (%) ↑	IDF1 (%) ↑	Inference Speed (FPS) ↑ RTX 3080Ti
BoT-SORT	51.77	80.41	1456
ByteTrack	51.85	80.38	1715
IoU-Greedy	56.76	74.47	5621
OC-SORT	54.88	72.68	4916
Proposed Method	74.59	93.24	1328

**Table 8:** Displacement-stratified performance of the proposed tracking method on the test set.

Test Subset	Clips	Frames	ACC (%)	IDF1 (%)
Overall	115	563	74.59	93.24
High Displacement (≥45.52)	51	244	72.76	90.20
Lower Displacement (<45.52)	64	319	79.93	95.06

**Table 9:** Performance comparison of different quantized models on the RK3588 platform.

Models	P (%)	R (%)	mAP50 (%)	Size (M)	Weight Memory (M)	Inference Time (ms)	Post_Process Time (ms)
yolo11n-normal_channel	98.72	98.72	97.60	4.3	2.59	23.33	0.36
yolo11n-normal_layer	98.79	98.70	97.56	4.3	2.56	23.72	0.38
yolo11n-IWAL_normal_layer	98.93	98.70	97.81	6.4	2.45	43.57	0.17
yolo11n-IWAL_normal_channel	98.99	98.72	97.91	6.4	2.49	43.75	0.17
yolo11n-IWAL_mmse_layer	98.93	98.57	97.72	6.4	2.45	44.49	0.17
yolo11n-IWAL_mmse_channel	98.96	98.67	97.84	6.4	2.49	44.44	0.18
yolo11n-IWAL_kl_layer	98.84	98.61	97.69	6.4	2.45	44.46	0.19
yolo11n-IWAL_kl_channel	98.95	98.68	97.88	6.4	2.49	44.57	0.18

**Table 10:** Inference performance comparison of the Siamese network with different quantization types on RK3588.

Quantization Type	Processing Time/ms (Avg. (Min–Max))				FPS ↑
	Preprocessing	Inference	Postprocessing	Pair Matching	
FP16	8.20 (5.00/144.80)	7.67 (5.24/16.38)	0.10 (0.05/0.65)	0.17 (0.13/0.41)	61.96
INT8	2.43 (1.12/139.66)	3.30 (2.38/16.30)	0.07 (0.04/0.53)	0.21 (0.13/0.68)	166.39
UINT8	2.87 (0.99/140.64)	3.60 (1.88/14.53)	0.08 (0.03/0.50)	0.25 (0.10/0.66)	147.06

## Data Availability

The datasets used in this study are not publicly available due to the confidentiality requirements of the dairy cow milking robot development project, but are available from the corresponding author upon reasonable request.
